# Silicon-Doped Titanium Dioxide Nanotubes Promoted Bone Formation on Titanium Implants

**DOI:** 10.3390/ijms17030292

**Published:** 2016-02-26

**Authors:** Xijiang Zhao, Tao Wang, Shi Qian, Xuanyong Liu, Junying Sun, Bin Li

**Affiliations:** 1 Department of Orthopaedics, the First Affiliated Hospital, Orthopaedic Institute, Soochow University, 188 Shizi St, Suzhou 215006, China; zhaoxijiangruilet@aliyun.com (X.Z.); wtfamily@163.com (T.W.); 2 Department of Orthopedics, the Affiliated Hospital of Jiangnan University, 200 Huihe Rd, Wuxi 214062, China; 3 State Key Laboratory of High Performance Ceramics and Superfine Microstructures, Chinese Academy of Sciences, 1295 Dingxi Rd, Shanghai 200050, China; qianshi@mail.sic.ac.cn

**Keywords:** silicon doping, titanium dioxide nanotubes, titanium, osteogenic differentiation, MC3T3-E1 cells, osseointegration

## Abstract

While titanium (Ti) implants have been extensively used in orthopaedic and dental applications, the intrinsic bioinertness of untreated Ti surface usually results in insufficient osseointegration irrespective of the excellent biocompatibility and mechanical properties of it. In this study, we prepared surface modified Ti substrates in which silicon (Si) was doped into the titanium dioxide (TiO_2_) nanotubes on Ti surface using plasma immersion ion implantation (PIII) technology. Compared to TiO_2_ nanotubes and Ti alone, Si-doped TiO_2_ nanotubes significantly enhanced the expression of genes related to osteogenic differentiation, including Col-I, ALP, Runx2, OCN, and OPN, in mouse pre-osteoblastic MC3T3-E1 cells and deposition of mineral matrix. *In vivo*, the pull-out mechanical tests after two weeks of implantation in rat femur showed that Si-doped TiO_2_ nanotubes improved implant fixation strength by 18% and 54% compared to TiO_2_-NT and Ti implants, respectively. Together, findings from this study indicate that Si-doped TiO_2_ nanotubes promoted the osteogenic differentiation of osteoblastic cells and improved bone-Ti integration. Therefore, they may have considerable potential for the bioactive surface modification of Ti implants.

## 1. Introduction

Taking advantage of their excellent biocompatibility and mechanical properties, titanium (Ti) and its alloys have been extensively used in orthopedic and dental applications these days [1,2]. However, the intrinsic bioinertness of untreated Ti surface usually results in insufficient bone formation and consequent osseointegration, which increases the risk of instability after surgical procedures including total joint replacement such as total hip arthroplasty (THA) or spinal fusion [3,4]. Instability after THA, for example, is a major cause of revision surgeries, contributing to 22.5% of THA revisions in the United States [5]. Therefore, a number of attempts have been made to improve the bioactivity of Ti surface in order to accelerate biological fixation of implants and reduce the chance of aseptic loosening of them.

In recent years, surface modification of Ti alone and Ti alloys by changing the surface chemistry [6,7], wettability [8], roughness [2,9], surface energy [10], microstructure [11,12] and nanotopography [13,14] has been tried to enhance their osseointegrative potential. Introducing anodic titanium dioxide (TiO_2_) nanotubes onto the Ti surface is among one of the approaches. A highly ordered TiO_2_ nanotube array with the lateral dimension within the sub-100 nm region forms on the surface of Ti substrate when it is anodized in fluoride-containing electrolytes [15,16]. TiO_2_ nanotubes have proven effective in promoting bone formation. For example, the growth of osteoblasts was accelerated by as much as 300%–400% when grown on anodic TiO_2_ nanotubes compared to the osteoblasts grown on unanodized Ti [17]. Cells grown on TiO_2_ nanotube surfaces also showed stronger ALP activity and more calcium deposition [18,19].

In addition to surface topography, different elements such as carbon [20], tantalum [21], zinc [22], magnesium [23], and silicon (Si) [24] have been introduced onto Ti surfaces through various surface treatment techniques for improving their osteogenic activity. A relatively less used trace element, Si, has been found to localize at the active sites of calcification in young bone and is therefore suggested to be closely related to calcium at early calcification stage [25]. In general, the introduction of a small amount of Si may markedly affect the adhesion, proliferation, and differentiation of osteoblastic cells and mesenchymal stem cells (MSCs) [26,27,28,29]. For example, Botelho *et al.* found that the expression of osteoblastic marker genes was up-regulated in cells grown on apatite substituted with 0.8 wt % Si [27]. In an *in vivo* study, Camiré *et al.* discovered 1 wt % Si-doped α-tri-calcium phosphate (α-TCP) showed significantly increased osteoclastic and osteoblastic activities and bone integration compared to pure α-TCP [30]. In contrast, Si deficiency may lead to abnormal bone formation [25,31].

Recently, we have found that, compared to pure TiO_2_, Si-doped TiO_2_ coatings prepared using cathodic arc deposition [32,33] or micro-arc oxidation [24] favored the activities of osteoblastic cells. In this study, we were interested in examining whether Si doping could further enhance the osteogenic potential of TiO_2_ nanotubes. To this end, we prepared three groups of Ti-based substrates, *i.e.*, Ti alloy (Ti), Ti alloy with TiO_2_ nanotubes at the surface (TiO_2_-NTs), and Si-doped Ti alloy with TiO_2_ nanotubes at the surface (Si–TiO_2_-NTs). We then compared the activities of MC3T3-E1 cells on them and also determined the *in vivo* fixation strength of implants made from the above materials through pull-out biomechanical tests.

## 2. Results

### 2.1. Preparation of TiO_2_-NTs and Si–TiO_2_-NT Substrates

**Figure 1:**
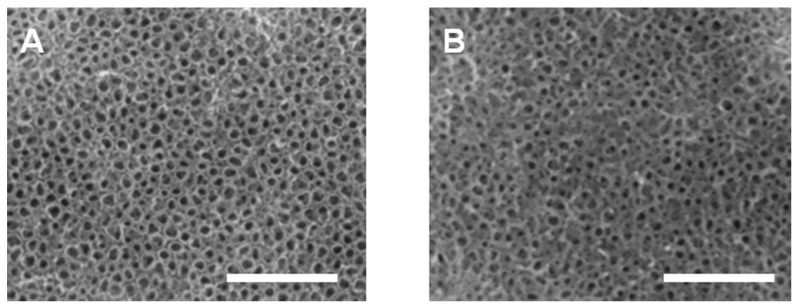
SEM images of TiO_2_-NTs (**A**) and Si–TiO_2_-NTs (**B**) surfaces. Scale bars, 1 μm.

**Figure 2:**
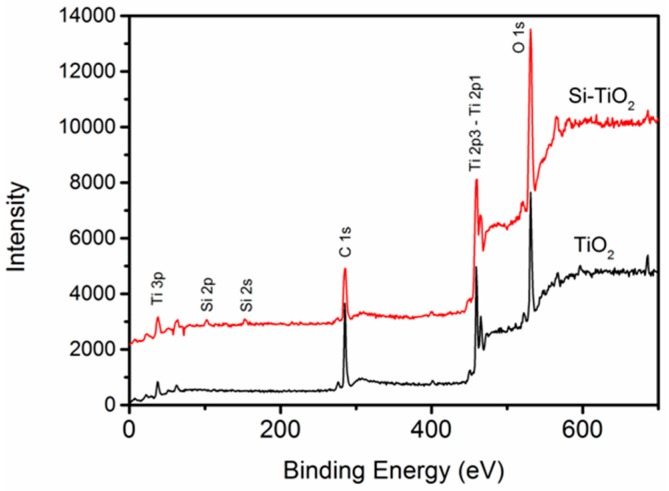
The chemical profiles of TiO_2_-NTs and Si–TiO_2_-NT surfaces determined using XPS.

TiO_2_-NTs were prepared via electrochemical anodic oxidation on the surface of Ti substrates. Following that, Si–TiO_2_-NT substrates were prepared using the Si plasma immersion ion implantation technique. As shown from SEM imaging (Figure 1), Si–TiO_2_-NTs had almost identical microstructures as TiO_2_-NTs, meaning that Si doping treatment did not cause apparent change of the surface topography of TiO_2_-NTs. The TiO_2_ nanotubes had an outer diameter of ~70 nm and inner diameter of ~60 nm. Figure 2 shows the characteristic peaks of Ti, O and C elements in the X-ray photoelectron spectroscopy (XPS) spectra of TiO_2_-NT and Si–TiO_2_-NT substrates. The characteristic peaks of Si were only seen on Si-treated substrates, indicating that Si had been successfully doped into the TiO_2_ nanotubes. The Si content was estimated to be about 0.51 wt %.

### 2.2. Cell Adhesion and Proliferation

**Figure 3:**
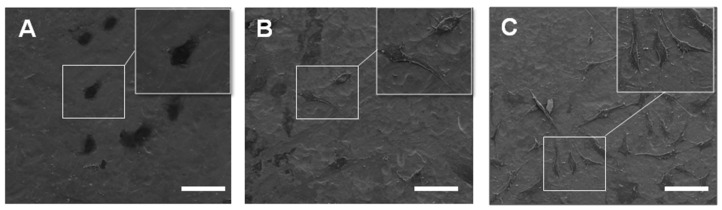
The morphology of MC3T3-E1 cells cultured on Ti (**A**); TiO_2_-NTs (**B**); and Si–TiO_2_-NTs (**C**) for 24 h as observed using SEM. Scale bars, 100 μm.

**Figure 4:**
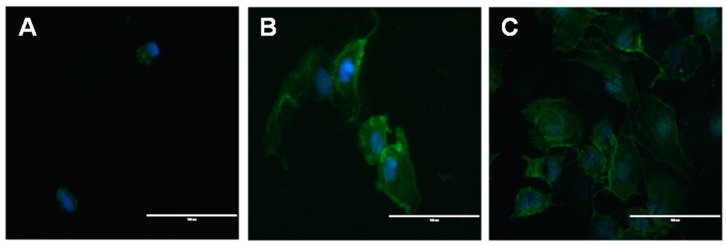
The immunofluorescence images of MC3T3-E1 cells that were stained with DAPI (**blue**) for nuclei and FITC-labeled phalloidin (**green**) for actin filaments after being cultured on (**A**); TiO_2_-NTs (**B**); and Si–TiO_2_-NTs (**C**) for 12 h. Scale bars, 100 μm.

Figure 3 shows SEM images of MC3T3-E1 pre-osteoblastic cells after 24 h of culture on Ti, TiO_2_-NT and Si–TiO_2_-NT substrates. As can be clearly seen, the cells showed relatively round morphology when cultured on Ti. However, they exhibited much flattened and irregular morphology on TiO_2_-NT and Si–TiO_2_-NT substrates. Moreover, extensive filopodial processes were seen on both TiO_2_-NT and Si–TiO_2_-NT substrates but not on Ti substrates. As shown by the immunofluorescence that revealed the cytoskeletal actin of cells, the cells on Ti remained small and round and seemed to lack microfilaments (Figure 4). The cells became markedly larger on TiO_2_-NT and Si–TiO_2_-NT substrates. Importantly, the actin filaments seemed to be better developed within MC3T3-E1 cells on Si–TiO_2_-NT substrates than on TiO_2_-NT substrates.

**Figure 5:**
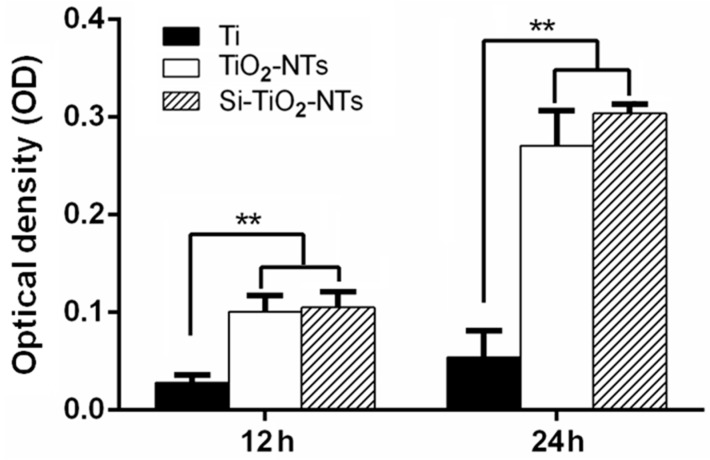
Proliferation of MC3T3-E1 cells on Ti, TiO_2_-NT and Si–TiO_2_-NT substrates. **, *p* < 0.01.

The proliferation of MC3T3-E1 cells cultured on Ti, TiO_2_-NT and Si–TiO_2_-NT substrates was measured via MTS assays (Figure 5). The number of cells on Ti substrates was obviously less than those on TiO_2_-NT and Si–TiO_2_-NT groups (*p* < 0.01). There appeared to be no statistically significant difference between the proliferation rates of cells on Si–TiO_2_-NT substrates and those on TiO_2_-NTs.

### 2.3. Osteogenic Gene Expression and Mineralization

**Figure 6:**
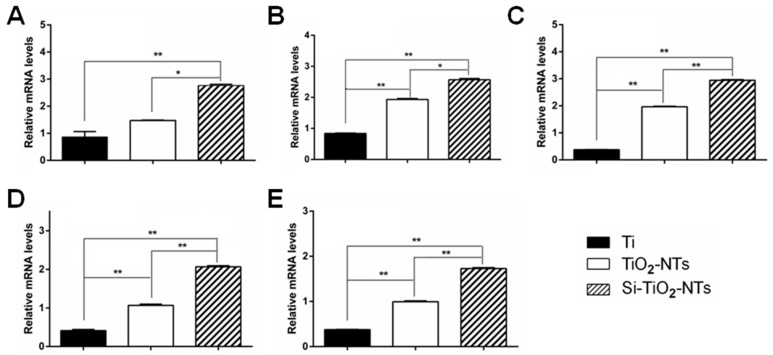
The expression of osteogenic differentiation related genes in MC3T3-E1 cells cultured on Ti, TiO_2_-NT, and Si–TiO_2_-NT substrates for seven days. (**A**) Col-I; (**B**) ALP; (**C**) Runx2; (**D**) OCN; and (**E**) OPN. *, *p* < 0.05; **, *p* < 0.01.

The expression of typical osteogenic differentiation-associated genes, including Col-I, ALP, Runx2, OCN, and OPN, in MC3T3-E1 cells was determined using real time PCR (Figure 6). Clearly, the expression of all above genes in the cells cultured on Si–TiO_2_-NTs for 7 days was significantly stronger than that of cells on Ti and TiO_2_-NT substrates (*p* < 0.01). Therefore, the osteogenic activity of cells was improved when they were cultured on Si–TiO_2_-NTs compared to both Ti and TiO_2_-NTs.

**Figure 7:**
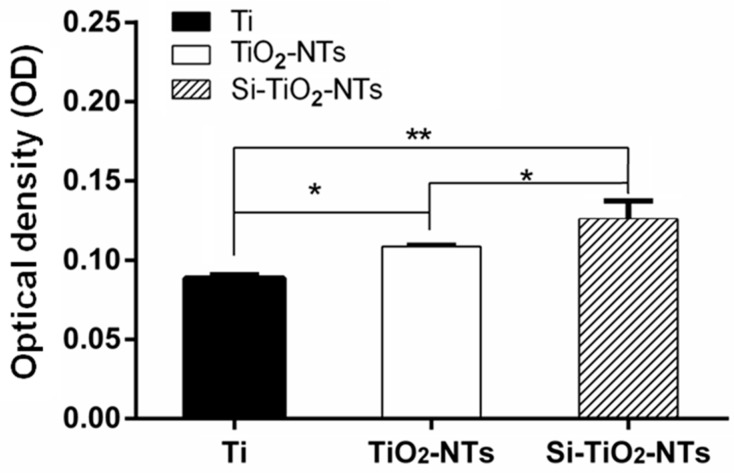
The extent of mineralization of MC3T3-E1 cells after 14 days of culture on Ti, TiO_2_-NT, and Si–TiO_2_-NT substrates. The samples were fixed and stained with alizarin red, which was then dissolved using 0.5 mol/L HCl and 5% SDS. The optical density of the solutions was measured as an indication of mineralization level. *, *p* < 0.05; **, *p* < 0.01.

Figure 7 shows the extent of calcium deposition of MC3T3-E1 cells after two weeks of culture. The amount of mineralization, shown by the mineral nodule formation, by the cells cultured on Si–TiO_2_-NT substrates was markedly higher compared to those on Ti and TiO_2_-NT substrates. In addition, the cells cultured on TiO_2_-NT substrates also had more calcium deposition than those on Ti substrates (*p* < 0.05).

### 2.4. Mechanical Tests

**Figure 8:**
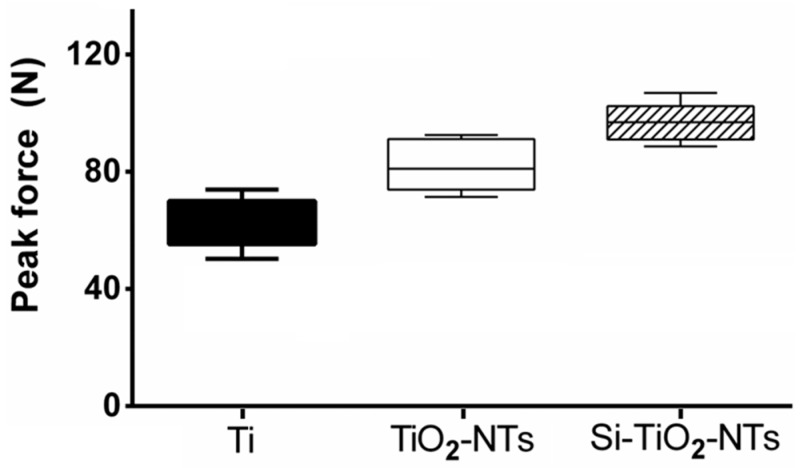
The peak pull-out forces upon removal of Ti, TiO_2_-NT, and Si–TiO_2_-NT screws after two weeks of implantation in rat femur (*p* < 0.05).

In order to check the integration between Ti-based substrates and bone tissue, pull-out tests were performed using Ti screws and Ti screws treated with TiO_2_-NTs or Si–TiO_2_-NTs. The screws were implanted in the femur of rats for two weeks prior to pull-out tests. The peak pull-out force indicates the strength of screw fixation which is affected by the implant-bone integration. Apparently, both Si–TiO_2_-NT and TiO_2_-NT screws resulted in stronger implant fixation than Ti screws (Figure 8). With the use of Si–TiO_2_-NT screws, the peak pull-out force was increased by 18% and 54% compared to the use of TiO_2_-NT and Ti screws, respectively (*p* < 0.05).

## 3. Discussion

Insufficient osseointegration is the major cause of orthopaedic implant loosening which leads to the failure of implants after total joint replacement or spinal fusion surgeries [3,4]. The surface chemistry and topography of implants are the most critical features that ultimately determine their ability to integrate with the surrounding bone tissue [34]. TiO_2_ nanotubes have been shown to enhance cell adhesion, proliferation, and differentiation [35,36,37]. Meanwhile, Si-substituted bioceramics promote osteogenesis and bone formation [27,30,38]. Therefore, it is anticipated that Si-doped TiO_2_ nanotubes may further enhance the growth activity and osteogenic potential of osteoblastic cells as a result of the synergistic effect of nanotubular microstructure and Si chemistry.

As expected, MC3T3-E1 cells showed more spread morphology and extended more filopodia when cultured on both Si–TiO_2_-NT and TiO_2_-NT substrates compared to those on Ti surfaces (Figure 3 and Figure 4). This is in accordance with a number of previous studies in which both surface micro-/nano-topography and Si doping were found to markedly affect cellular behaviors [32,39,40]. All substrates did not show any cytotoxicity. However, cells apparently proliferated faster on Si–TiO_2_-NT and TiO_2_-NT substrates compared to Ti (Figure 5). It is likely that the size of TiO_2_ nanotubes (70–100 nm) favored integrin clustering and focal contact formation of cells, thus providing adequate anchoring sites for the cells [41]. Further doping with Si did not bring in any negative effect.

Osseointegration is a late stage of the evolution of implant fixation, characterized by the establishment of bone-implant contact and peri-implant bone development following bone matrix mineralization [42]. As seen, the expression of typical osteogenic genes (Col-I, ALP, Runx2, OCN and OPN) was markedly up-regulated in MC3T3-E1 cells cultured on Si–TiO_2_-NT substrates compared to those on Ti and TiO_2_-NTs (Figure 6). Among them, Col-I is the major component which makes up over 90% of the organic bone matrix, ALP represents a specific subset of markers of osteoblast phenotype, Runx2 is the main transcription factor required for the osteoblast lineage commitment of multipotent mesenchymal cells, yet OCN and OPN indicate the onset of mineralization [43,44]. In addition, Si–TiO_2_-NT substrates promoted the formation of minerals better than TiO_2_-NT and Ti substrates (Figure 7). Therefore, although there was no marked difference between the cell proliferation on Si–TiO_2_-NT and TiO_2_-NT substrates, the osteogenic activity of cells was markedly improved when they were cultured on Si–TiO_2_-NT compared to both TiO_2_-NT and Ti substrates. The fact that Si doping affected the differentiation of cells, but not their proliferation, on TiO_2_-NT treated surfaces slightly differs from our previous finding, in which Si-doped TiO_2_ films appeared to favor both osteoblastic proliferation and differentiation [32]. This might be the result of the topographical difference (rough TiO_2_ nanotubes *versus* relatively smooth TiO_2_ film surface).

The enhanced osteogenic capacity of Si–TiO_2_-NT substrates is a very important attribute for *in vivo* implantation as it allows more new bone formation on the implant surface and therefore strengthens the interlocking between implant and bone tissue. Indeed, we found significantly increased implant fixation strength with Si–TiO_2_-NT implants after an implantation period as short as two weeks (Figure 8). The peak pull-out force of Si–TiO_2_-NT implants was not only substantially greater than that of Ti implants (54% higher), but also markedly larger than that with TiO_2_-NTs (18% higher). Such biomechanical test results correlate well with the gene expression and mineralization results (Figure 6 and Figure 7), implying that the increased matrix deposition and mineralization around Si–TiO_2_-NT screws significantly enhanced the osseointegration of implants.

## 4. Materials and Methods

### 4.1. Materials

Ti (99.6% purity) in the form of discs with diameter of 5.8, 13 and 31 mm, respectively, and thickness of 1 mm were used in this study. For *in vivo* experiments, pure Ti screws, which were 10 mm long and had outer thread diameter of 2.0 mm and inner thread diameter of 1.7 mm, were used. The material was supplied by Tianjin Zhengtian Medical Device Company (Tianjin, China).

### 4.2. Fabrication of TiO_2_ Nanotube-Coated Ti Substrates

Pure titanium screws and discs were ultrasonically cleaned using ethanol and deionized water consequently before being treated using 5 wt % oxalic acid solution at 100 °C for 2 h [45]. Following that, the pretreated specimens were anodized at a constant direct current (DC, 20 V) for 30 min in an electrolyte composed of 1.0 wt % hydrofluoric acid. After the anodizing oxidation, the specimens were rinsed with distilled water and air-dried. The morphology of TiO_2_ nanotubes on the surface of specimens was characterized by field emission scanning electron microscopy (SEM) (S-4800, Hitachi, Tokyo, Japan).

### 4.3. Si Plasma Immersion Ion Implantation (Si-PIII)

Si was implanted into the TiO_2_ nanotubes using a filtered cathodic arc plasma source. In brief, a curved magnetic duct was inserted between the plasma source and main chamber for removing the macro-particles generated by the cathodic arc. The cathode rod, 10 mm in diameter, was made of 99.99% pure metallic Si. The discharge of Si ions was regulated by the main arc current between the cathode and anode. By applying a pulsed high voltage to the TiO_2_ nanotubes samples, Si ions were implanted. The ion implantation time was 0.5, 1, 1.5 h and the pressure was 2.5 × 10^−3^ Pa. During Si-PIII, the main arc current and the pulsed high voltage that was applied to the target were synchronized at a frequency of 6 Hz. The pulse duration of high voltage was set at 450 ms. During Si-PIII, the sample stage was actively cooled by circulating water to keep the sample temperature at 25 °C.

### 4.4. Surface Characterizations

The morphology of sample surfaces was examined using SEM (S-3400, Hitachi, Tokyo, Japan). The surface chemistry and elemental depth profiles were determined using XPS (PHI 5802, Physical Electronics, London, UK). The Si, C, O, and Ti profiles were acquired by XPS in conjunction with argon ion bombardment at a sputtering rate of about 4 nm/min.

### 4.5. Cell Culture

MC3T3-E1 mouse preosteoblasts (CRL-2594, subclone 14, ATCC) were cultured in α- mininum essential medium (α-MEM, Gibco, Grand Island, NY, USA) supplemented with 10% fetal bovine serum (Gibco) and 1% penicillin/streptomycin at 37 °C in a 5% CO_2_ environment. Cells were seeded onto different experimental substrates. The plates with dimensions 5.8 mm were placed on 96-well polystyrene plates. The cells were seeded onto the specimen substrate at an initial density of 1 × 10^4^ cells/well. The 13 mm plates were placed on 24-well polystyrene plates and the concentration was 3 × 10^4^ cells/well. The 31 mm plates were placed on 6-well polystyrene plates and the concentration was 3 × 10^5^ cells/well. In the osteogenic differentiation assay, after the cells were cultured for 24 h, the medium was further supplemented with β-glycerol phosphate (10 mM), ascorbic acid (50 μg/mL), and dexamethasone (10 nM) for osteogenic induction. The media were refreshed every 3 days.

### 4.6. Cell Morphology

The cells on surfaces with a diameter of 5.8 mm were cultured 24 h. After washed with PBS, the samples were fixed using 2.5% glutaraldehyde (Sigma, St. Louis, MO, USA) in PBS for 1 h and then rinsed three times with PBS for 10 min. After that, the cells were dehydrated in a graded series of ethanol (30%, 50%, 70%, 90% and 100%) for 30 min each and left in 100% ethanol. The samples were dried using a Critical Point Dryer (CPD030, LEICA, Wetzlar, Germany) and sputter-coated with gold using an Ion Sputter (SC7620, Quorum Technologies, Lewes, UK). Finally, the morphology of cells was observed by SEM (Quanta 250, FEI, Hillsboro, OR, USA).

### 4.7. Immunofluorescence of Cytoskeletal Actin

After culturing for 24 h, MC3T3-E1 cells were fixed using 4% paraformaldehyde in PBS for 15 min at room temperature. The cells were then washed twice with PBS and permeabilized with Triton X-100. After that, they were incubated with FITC-labeled phalloidin (5 µg/mL in PBS) containing 1% BSA for 1 h at room temperature. Then, the cell nuclei were stained with DAPI. The cells were washed three times with PBS for 5 min each wash. Cell morphology and cytoskeletal arrangement were observed using an EVOS fluorescence microscope (AMG, Thornwood, NY, USA).

### 4.8. MTS Assay

Cell proliferation was evaluated using MTS assay. Samples with 5.8 mm in diameter were placed in a 96-well plate. The concentration was 1 × 10^4^ cells/well. Cells were cultured for 12 and 24 h, respectively, at 37 °C and 5% CO_2_. At predetermined periods, samples were washed three times with PBS and transferred to a new 96-well polystyrene culture plate. After that, 100 °C culture medium and 20 μL MTS reagent (Promega, Madison, WI, USA) were added to each well following the manufacturer’s directions. After 2 h of incubation under 5% CO_2_ atmosphere, the absorbance at a wavelength of 490 nm of each solution was measured using a 96-well plate reader.

### 4.9. Mineralization Assay

The degree of osteogenesis was evaluated by Alizarin Red S (Sigma) staining. After osteogenic induction for 2 weeks, three experimental surfaces with 13 mm in diameter were fixed in 75% ethanol for 1 h. The cells were rinsed with distilled water and stained using 200 μL of 40 mmol/L Alizarin red S (pH 4.1) solution for 10 min at room temperature. Afterwards, the unbound stain was totally removed by rinsing with double-distilled water. Quantitative analysis of Alizarin Red S staining was performed through solubilization with 0.5 mol/L HCl and 5% sodium dodecyl sulphate (SDS) for 30 min at room temperature [46]. Then, 100 μL of the solubilized stain was determined by the absorbance at 405 nm measured using a spectrophotometer (U-3010, Hitachi, Tokyo, Japan).

### 4.10. Quantitative Real-Time Polymerase Chain Reaction (qRT-PCR)

MC3T3-E1 cells were first seeded on three experimental specimens 31 mm in diameter in 6-well plates at the density of 3 × 10^5^ cells/well. After culturing for 24 h, 10 mM β-glycerol phosphate, 50 μg/mL ascorbic acid, and 10 nM dexamethasone were added for osteogenic induction. After culturing with cells for 7 days, the three experimental specimens were rinsed three times with PBS, and the total RNA of cells was extracted with Trizol (Sigma). One microgram of total RNA was reverse transcribed into cDNA using SuperScript III First-Strand Synthesis System (Invitrogen, Carlsbad, CA, USA) according to the manufacturer’s protocols. qRT-PCR was performed to evaluate the expression of Col-I, Runx2, ALP, OCN, and OPN. All samples were analyzed in triplicates. Data analysis was carried out using the iQ5 Optical System Software Version 2.0 (BioRad, Berkeley, CA, USA). The relative expression level (fold change) was calculated by converting the logarithmic values into absolute values using 2^−ΔΔ*C*t^. GAPDH was used as the housekeeping gene. The primer sequences of the genes are shown in Table 1.

### 4.11. Animal Surgeries and Mechanical Tests

Nine male SD rats (weighing 260–280 g) supplied by Soochow University Animal Center were used for the animal tests. They were randomly divided into three groups according to the implants, *i.e.*, pure titanium screws (Ti), titanium screws subjected to anodic oxidation treatment (TiO_2_-NTs), and titanium screws subjected to anodic oxidation and silicon plasma ion implantation treatments (Si–TiO_2_-NTs). Systemic anesthesia was performed using ketamine and xylazine at 80–100 and 10–20 mg/kg, respectively. In addition, local anesthesia was also performed at the surgery area using 2% lidocaine solution containing epinephrine (1:100,000). The hair of surgical site was shaved and sterilized with betadine scrubs. Flat surfaces of the distal femurs were selected for implant placement. The screws were implanted from the outside to the inside in the horizontal direction. Each rat received one implant in each femur. After 2 weeks, the experimental animals were sacrificed by an excessive intraperitoneal dose of sodium pentobarbital. Pull-out tests were performed for all implants (6 implants per group) in the femur. The screws were tested for pull-out strength using a materials testing system (HY-1080, Shanghai, China) at a test speed of 1 mm/s. The maximal force was considered as the pull-out force. All surgeries were performed according to the animal surgery protocol approved by the Institutional Animal Care and Use Committee (IACUC) of Soochow University. (Approval Date: 3 March 2014) All measurements were performed while the investigators were blinded for treatment.

### 4.12. Statistical Analysis

Each experiment was performed three times and the results were showed as mean ± standard deviation. All data were analyzed using SPSS 13 software, with *p* < 0.05 being considered statistically significant. The graphs show averages ± standard error bars and the significance between groups is marked on the graphs.

## 5. Conclusions

In this study, Ti substrates that were decorated with TiO_2_ nanotubes at the surface and further doped with Si using plasma immersion ion implantation technology have been prepared. It was found that TiO_2_-NT and Si–TiO_2_-NT substrates similarly promoted the adhesion and proliferation of pre-osteoblastic MC3T3-E1 cells compared to Ti alone. However, the osteogenic activities of cells, including the expression of genes associated with osteogenic differentiation and the matrix mineral deposition, were markedly enhanced on Si–TiO_2_-NT substrates compared to TiO_2_-NTs. After two weeks of implantation in rat femur, the pull-out tests showed that the fixation strength of Si–TiO_2_-NT implants was 18% and 54% higher compared to TiO_2_-NT and Ti implants, respectively. Therefore, the synergetic effect of surface chemistry and nanostructured topography may predict substantially enhanced osteogenic potential of Ti substrates and improved bone-Ti integration, which is critical toward optimal orthopedic implants. Further studies will explore the specific mechanisms by investigating the effect of Si concentration at implant surface as well as comparative analysis of protein adsorption and biomolecule interactions on Si–TiO_2_-NT and TiO_2_-NT surfaces.

## Figures and Tables

**Table 1:** The sequences of the oligonucleotide primers for quantitative real-time polymerase chain reaction (qRT-PCR) assays.

Gene	Primers Sequence	Amplicon Size (bp)	Accession Number
Col-I	Forward: 5′-CCTGAGTCAGCAGATTGAGAACA-3′	114	NM_007742
	Reverse: 5′-CCAGTACTCTCCGCTCTTCCA-3′		
OC	Forward: 5′-CGCTCTGTCTCTCTGACCTC-3′	91	NM_001037939
	Reverse: 5′-CACTACCTTATTGCCCTCCTG-3′		
OPN	Forward: 5′-CTTTCACTCCAATCGTCCCTAC-3′	99	NM_001204201
	Reverse: 5′-CAGAAACCTGGAAACTCCTAGAC-3′		
ALP	Forward: 5′-GGGCATTGTGACTACCACTCG-3′	103	NM_001287172
	Reverse: 5′-CCTCTGGTGGCATCTCGTTAT-3′		
Runx2	Forward: 5′-GACACTGCCACCTCTGACTT-3′	115	NM_001145920
	Reverse: 5′-GATGAAATGCTTGGGAACTG-3′		
GAPDH	Forward: 5′-CATCAAGAAGGTGGTGAAGC-3′	198	NM_001289726
	Reverse: 5′-CCTGTTGCTGTAGCCGTATT-3′		

## Abbreviations

Ti: Titanium
Si: Silicon
TiO_2_: Titanium dioxide
PIII: Plasma immersion ion implantation
THA: Total hip arthroplasty
MSCs: Mesenchymal stem cells
α-TCP: α-Tri-calcium phosphate
SEM: Scanning electron microscopy
XPS: X-ray photoelectron spectroscopy
α-MEM: α-Mininum essential medium
SDS: Sodium dodecyl sulphate
IACUC: Institutional Animal Care and Use Committee
